# Evaluating physicians’ awareness and prescribing trends regarding proton pump inhibitors: a cross-sectional study

**DOI:** 10.3389/fphar.2023.1241766

**Published:** 2023-11-09

**Authors:** Sarya Swed, Hidar Alibrahim, Haidara Bohsas, Ahmed R. N. Ibrahim, Abdelmonem Siddiq, Nagham Jawish, Mark Hasib Makhoul, Maram Abdulmajid Mahmoud Alrezej, Fouad Hasib Makhoul, Bisher Sawaf, Wael Hafez, Sarah Makram Elsayed, Rami Soliman, Engy A. Wahsh

**Affiliations:** ^1^ Faculty of Medicine, Aleppo University, Aleppo, Syria; ^2^ Department of Clinical Pharmacy, College of Pharmacy, King Khalid University, Abha, Saudi Arabia; ^3^ Faculty of Pharmacy, Mansoura University, Mansoura, Egypt; ^4^ Faculty of Medicine, Damascus University, Damascus, Syria; ^5^ Faculty of Pharmacy, Al Baath University Homs, Homs, Syria; ^6^ Faculty of Medicine, Al Baath University Homs, Homs, Syria; ^7^ Department of Internal Medicine, Hamad Medical Corporation, Doha, Qatar; ^8^ NMC Royal Hospital, Abu Dhabi, United Arab Emirates; ^9^ Medical Research Division, Department of Internal Medicine, The National Research Centre, Cairo, Egypt; ^10^ Faculty of Medicine, October 6 University, Giza, Egypt; ^11^ National institute of Chest and Allergy, Egypt - Mediclinic Hospital, Abu Dhabi, United Arab Emirates; ^12^ Department of Clinical Pharmacy, Faculty of Pharmacy, October 6 University, Giza, Egypt

**Keywords:** adverse event (AE), physician, proton pump inhibitors, Syria, upper gastrointestinal (GI) bleeding

## Abstract

**Introduction:** Proton pump inhibitors (PPIs) are commonly used to treat acid-related disorders. Their appropriate use depends on the correct indications from the clinician. Owing to the high incidence of use and misuse, PPIs have been identified as an essential pharmacological class for developing deprescribing recommendations. Therefore, assessing physicians’ knowledge and practice regarding PPI usage is critical for paving the way toward targeted recommendations and efforts.

**Objective:** This study aimed to assess Syrian physicians’ perceptions of proton pump inhibitors adverse effects, their benefit in upper gastrointestinal bleeding (UGIB) prophylaxis, and how these perceptions are related to PPI prescription practice.

**Methods:** A cross-sectional study was performed using a web-based questionnaire distributed among Syrian physicians in internal medicine between 28 November and 23 December 2022. The questionnaire assessed perceptions and experiences of PPIs, concerns about specific adverse effects, and their effectiveness for UGIB prophylaxis, in addition to the different scenarios used to determine the best practice for appropriate treatment to manage minimal, mild, moderate, and high-risk UGIB patients.

**Results:** A total of 473 participants completed the questionnaire, with median age ±SD was (28.46 ± 4.58), and most participants (83.3%) were residents. Approximately half of the participants (45.5%) agreed that discussion assistance was provided to continue or terminate PPIs properly. Only 8.9% were very familiar with published evidence of PPI adverse effects. Bone weakening and vitamin B12 deficiency were the most frequently reported side effects (81.8% and 79.7%, respectively). However, dementia (0.4%) and mortality (1.9%) were the least reported adverse effects. More than half of the participants (64%) perceived using PPIs to prevent upper GI bleeding. Non-trainee physicians were less knowledgeable about appropriate GERD management than resident physicians (*p* < 0.001).

**Conclusion:** The study showed a gap between Syrian physicians’ perceptions and practices regarding PPI use, which necessitates spreading awareness of updated guidelines for PPI usage and their side effects.

## 1 Introduction

Since the advent of omeprazole in 1989, proton pump inhibitors (PPIs) have progressively replaced the traditional therapies for acid-related diseases. PPI use has grown extremely prevalent among primary care doctors and is now a staple component of the gastroenterologist’s repertoire (Strand et al., 2017). These drugs constitute the first line of defense against esophagitis, non-erosive reflux disease, peptic ulcer disease, NSAID-induced ulcers, Zollinger-Ellison syndrome, and functional dyspepsia (Chiba et al., 1997; Shi and Klotz, 2008). In addition to antibiotics, it is considered an essential component of *Helicobacter pylori* eradication treatment (Klotz, 2000). In the United States, PPIs are among the most frequently used drugs (List of Top 50 Prescription Drugs Filled in the US, 2016). PPIs are often prescribed or used for long periods, without reference or instruction (Metaxas and Bain, 2015; Mafi et al., 2019). A meta-analysis of 23 trials with over 300,000 participants found a 65% increase in the prevalence of *Clostridium* difficile-associated diarrhea among those who used PPI (Janarthanan et al., 2012). Another research of 11,280 people with *Salmonella*, *Campylobacter*, and other enteric infections found that acid suppression was associated with an increased risk, with a higher association with PPI than with H2-receptor antagonists (Leonard et al., 2007). The majority of common illnesses, such as gastroesophageal reflux disease (GERD), require only short-term treatment (approximately 4–8 weeks) (Ramakrishnan and Salinas, 2007; Katz et al., 2013). According to studies, 40% and 65% of hospitalized patients in the United States and Australia lack a proven ongoing rationale for taking it, implying that continuous use may be harmful (Heidelbaugh et al., 2010; Heidelbaugh et al., 2012). Owing to their high incidence of use and misuse, PPIs have been identified as an essential pharmacological class for developing deprescribing recommendations using a countrywide modified Delphi consensus method (Farrell et al., 2015). Choosing Wisely, which is an initiative of The American Board of Internal Medicine Foundation, in 2012, spread information by minimizing the widespread PPI usage by advising the proper prescription, dose, and duration in treating hurt burns and GERD (Treating Heartburn and GERD, 2012). A 2013 internist study found that they often incorrectly advised patients to discontinue PPIs while being used to treat GERD or prevent upper gastrointestinal bleeding (UGIB) (Kurlander et al., 2017). The results of the study highlight the need to carefully consider a patient’s medical history, symptoms, and current medications before deprescribing. PPIs have been linked to a variety of serious disorders, including dementia, chronic kidney disease, and a higher risk of death; fresh evidence supporting PPI deprescribing has also been released (Gomm et al., 2016; Lazarus et al., 2016; Farrell et al., 2017; Xie et al., 2017). It is unknown how doctors perceive the hazards of PPIs and whether they are altering their prescribing and deprescribing practices appropriately. Our cross-sectional study aimed to assess Syrian clinicians’ perspectives on PPI adverse events (AEs), the benefits of PPIs in UGIB prophylaxis, and how these perceptions are related to PPI prescription practice.

## 2 Materials and methods

### 2.1 Study design and setting

From November 28 to 23 December 2022, nationwide cross-sectional research was conducted in Syria to investigate Syrian physicians’ perceptions of the risks of proton pump inhibitors. Internal medicine doctors, residents, and fellows in general practice or any medical specialty were eligible. Medical students, nursing personnel, other healthcare employees, non-Syrian physicians, and individuals who were unable to complete the survey were excluded. This questionnaire was developed using data from the University of Michigan’s Center for Bioethics and Social Sciences in Medicine, which comprises academics with experience in risk communication and decision making (Kurlander et al., 2020). The questionnaire was translated into Arabic and revised by an experienced healthcare provider. It was translated into English to ensure its correctness. We used convenience and snowball tactics to acquire information from respondents. A Google Form questionnaire was created and sent to respondents using social media sites such as Facebook, WhatsApp, and Telegram. Throughout Syria, hospitals, clinics, and other health facilities were open for data collection. Participation in this survey was voluntary, and participants were asked if they agreed to participate in this study before completing the questionnaire.

### 2.2 Sample size calculation

Sample size was calculated using Calculator.net (https://www.calculator.net/sample-size-calculator.html). According to the most recent Syrian Ministry of Health data (https://www.moh.gov.sy/), there were approximately 282,141 medical residents and fellows. We used the following criteria in a statistical power analysis to calculate the optimal sample size: a population percentage of 50%, a margin of error of 0.05, and a confidence level of 95%. The suggested sample size was 384.

### 2.3 Measures

#### 2.3.1 Sociodemographic variables and work-related characteristics

We inquired about general demographic, professional, and practice characteristics; familiarity with guidelines for the responsible use of PPIs; and the availability of decision support to aid with the sensible use or termination of PPIs.

#### 2.3.2 General familiarity, concern about possible PPI AEs, and awareness and beliefs about PPIs

The questionnaire asked about general awareness and concerns about possible PPI adverse events and knowledge of and views regarding whether PPIs may increase the risk of any of the 12 conditions associated with PPIs. Participants were asked about possible AEs from PPI administration they were most worried about. We also questioned how often patients who take PPIs express concerns about AEs, and how frequently clinicians address the benefits and drawbacks of PPIs before prescribing them. We also inquired about the extent to which AEs research changed practitioners’ PPI prescription habits.

#### 2.3.3 Clinical scenarios

Four typical clinical situations were given to the participants, including a 70-year-old female patient who took omeprazole 20 mg daily and had just been diagnosed with osteopenia, which would increase the risk of bone fracture, a condition associated with PPIs (Zhou et al., 2016). The degree of UGIB risk for the patients varied [minimal (history of GERD), low (low-dose aspirin), moderate (low-dose aspirin and warfarin), and high (prior peptic ulcer disease (PUD) and low-dose aspirin)]. After each scenario, we asked the participants how they would treat the patient’s PPI, providing the following answer options: continue taking omeprazole, discontinue omeprazole, and replace it with an H2-blocker. Randomization was used to determine how the situations were presented to the participants. Following the high-risk UGIB prevention scenario, participants were asked to assess the efficacy of omeprazole in decreasing UGIB risk on a Likert-type scale (response options: not at all, slightly, moderately, and very). Our UGIB risk evaluations for each scenario, which were not disclosed to survey respondents, are based on earlier risk assessments (García Rodríguez et al., 2011; Lanas et al., 2013). Without PPI, the survey’s low-, medium-, and high-risk UGIB scenarios were expected to carry 0.5%, 1.5%, and 2.7% annual chances of UGIB, respectively. Recent recommendations on appropriate PPI cessation (Farrell et al., 2017) would support PPI discontinuation in the event of GERD. The moderate-risk and high-risk GI bleeding scenarios, but not the low-risk scenario, are supported by PPI gastroprotection guidelines. The questionnaire was provided as Supplementary Material.

### 2.4 Pilot sample

The questionnaire was randomly distributed to Syrian clinicians to determine their level of clarity and readability. Some modifications were made based on the feedback provided by the participants. A pilot test was conducted with 25 physicians to evaluate the survey’s validity. High levels of internal consistency (Cronbach’s alpha varied from 0.712 to 0.861) were demonstrated before the questionnaire distribution.

### 2.5 Ethical consideration

Ethical approval was obtained from the Aleppo University Ethics Committee (IRB-LN/23-7), and all experiments were performed in accordance with the Declaration of Helsinki. Participants were given a URL to access the questionnaire via Google. Before answering the questionnaire, they were asked about their willingness to participate in the study and to complete the questionnaire. Responses were collected securely from an online database. All participants were informed of the study’s purpose, the research group’s name, their right to withdraw from the study, the confidentiality of their personal information, and that only fully provided information would be analyzed.

### 2.6 Statistical analysis

Descriptive statistics were computed for categorical variables such as frequency and percentage. We merged the categories of osteoporosis/osteopenia and bone fracture under the heading “bone loss or fracture” to calculate the frequency of knowledge and perceptions regarding AEs.

In the high-risk UGIB preventive scenario, we performed an exploratory multivariable analysis to assess the independent factors for continuing PPI. This scenario was chosen because it featured the highest indication for PPI continuation and a significant number of participants decided to stray from that approach. Since H2-blockers are likely inferior to PPIs for UGIB prevention and are not advised for this purpose, the dichotomous outcome was defined as PPI continuation vs. (stopping PPI or switching to an H2-blocker) (“PPI discontinuation”). Concern about PPI AEs, perceived PPI efficacy for avoiding UGIB, age, sex, trainee status, number of patients seen per week, familiarity with PPI usage recommendations for UGIB prevention, and availability of decision assistance for optimal PPI use were included as predictors. Concerns regarding PPI adverse effects and the perceived efficacy of PPIs for UGIB prevention were examined as four-level indicator variables, with “not at all” as the base category. We merged two levels of the scale for PPI effectiveness (not at all effective and slightly effective) into a single level for the regression because of the presence of complete separation of one of the predictors (PPI effectiveness) by the outcome variable, which precluded the maximum likelihood estimation of the model. Logistic regression was used to analyze the bivariate relationship between the predictor variables and clinical scenario management, as well as the final multivariable model. We expected that concern about AE and perceived efficacy for bleeding prevention would be adversely and favorably linked with recommending PPI continuation. Four examples were removed from this model due to missing age data.

## 3 Results

### 3.1 Sociodemographic characteristics

As shown in Table 1, among the 473 participants in this study, 51.2% were men, and the mean age ±SD was (28.46 ± 4.58), with the majority (81.8%) being between the ages of 25 and 29. The majority of the participants (83.3%) were residents. Most participants (23.3%) said they care for 1 to 25 patients every week. More than half of the participants (65.5%) reported that they followed up with 1–25 PPI patients every week. 45.5% Of participants dedicated 50%-74% of their time to patient care. Governmental health hospital staff made up 47.4% of the study sample, and two-thirds (64.1%) were acquainted with PPI recommendations to prevent upper GI bleeding. A total of 45.5% of the participants agreed that discussion assistance was provided to assist with the proper continuation or termination of PPIs.

**TABLE 1:** Participants’ demographics.

Variable	Categories	N (%)
Age (mean ± SD)		(28.46/4.586)
Age	25–29	387 (81.8)
	30–39	63 (13.3)
	40–49	18 (3.8)
	50–60	5 (1.1)
Gender	Male	247 (51.2)
	Female	226 (47.8)
Training level	Residency	394 (83.3)
	Non-trainee physician	21 (4.4)
	Fellowship	58 (12.3)
See outpatients in clinic	No	73 (15.4)
	Yes	400 (84.6)
Patients seen per week	None	44 (9.3)
	1–25	110 (23.3)
	26–50	104 (22)
	51–75	68 (14.4)
	76–100	102 (21.6)
	>100	45 (9.5)
Patients seen per week on PPI	None	43 (9.1)
	1–25	310 (65.5)
	26–50	89 (18.8)
	51–75	27 (5.7)
	76–100	3 (0.6)
	>100	1 (0.2)
Time spent on patient care	<25%	57 (12.1)
	25%–49%	127 (26.8)
	50%–74%	215 (45.5)
	75%–100%	74 (15.6)
Practice type	Military or other government-employed	5 (1.1)
	Group practice	19 (4)
	Independent Practice	33 (7)
	Hospital employed—Government health system	224 (47.4)
	Hospital employed—Private health system	21 (4.4)
	Academic practice	171 (36.2)
Familiar with guidelines on the appropriate use of PPIs to prevent upper GI bleeding	No	170 (35.9)
	Yes	303 (64.1)
Decision support is available to help with the appropriate continuation or discontinuation of PPIs	No	258 (54.5)
	Yes	215 (45.5)
Do you personally take a PPI at least once a week?	No	331 (70)
	Yes	142 (30)

### 3.2 Perceptions and experiences of PPIs

Only 8.9% were very familiar with published evidence on PPI adverse effects, and 64.3% had modified their PPI prescription patterns, either very much or somewhat, based on research on their adverse effects. When prescribing PPIs, a small fraction (7.2%) was unconcerned about side effects. Before providing PPIs, 41.6% of participants said that they rarely addressed the risks of adverse effects with patients. However, 30.4% said that their patients sometimes had repeated worried about the potential of PPI side effects (Table 2).

**TABLE 2:** Perceptions and experiences of PPIs among participants.

Variable	N (%)				
	Very much	Somewhat	Slightly	Not at all	Missing
Familiar with published data on PPI adverse effects	42 (8.9)	206 (43.6)	204 (43.1)	19 (4)	2 (0.4)
Have changed PPI prescribing habits as a result of studies on adverse effects	147 (31.1)	157 (33.2)	142 (20)	9 (1.9)	18 (3.8)
Concerned about adverse effects when prescribing PPIs	57 (12.1)	174 (36.8)	208 (44)	34 (7.2)	_
	Often	Sometimes	Rarely	Never	Missing
frequently discussing the risks of adverse effects with patients before starting a PPI	56 (11.8)	173 (36.6)	197 (41.6)	47 (9.9)	_
Recurring concerns about the risk of adverse effects from PPIs	11 (2.3)	144 (30.4)	231 (48.8)	87 (18.4)	_

### 3.3 Concern about specific adverse effects

**FIGURE 1:**
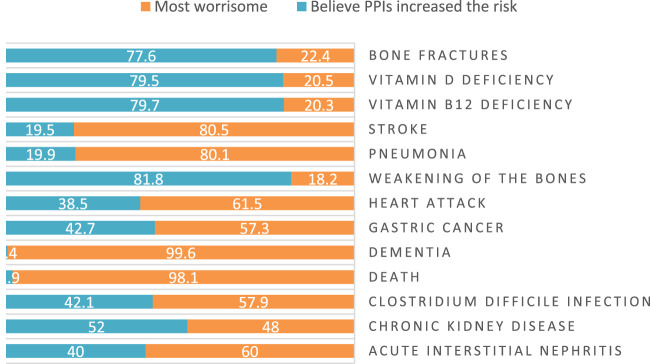
Participants beliefs about PPI related side effects.

When asked to rate 13 PPI-related side effects, bone weakening was the most frequently reported (81.8%), followed by vitamin B12 shortage (79.7%) and vitamin D deficiency (79.5%). Dementia (0.4%) and mortality (1.9%) were the least prevalent adverse consequences that individuals were aware of. The most concerning side consequence reported by participants was dementia (99.6%), followed by death (98.1%) (Figure 1).

### 3.4 Management of the patient scenarios and perceived effectiveness of PPIs for UGIB prevention

Three factors were significantly related to patient management in the GERD scenario (*p*-value <0.05). In the first case, 86.8% of resident physicians could pick the best treatment for with GERD patient. In the first scenario, the majority of participants (86%) who spent 50%–74% of their time in patient care, identified the best management for the patient.

Seven factors were significantly associated with patient treatment in the second scenario (*p* < 0.05). In the second scenario, 16.3% (n = 65) of the participants caring for outpatients were unable to select the appropriate therapy for the patient. In the second scenario, most responders (91.1%), who cared for more than 100 patients each week, chose real patient management.

In the third scenario, we discovered a significant association between these six factors and patient treatment (*p* < 0.05). In the third scenario, the majority (91.9%) of participants who spent 75%–100% of their time caring for patients did not choose the proper therapy. In the third scenario, only a small number of respondents (8.9%) who worked in government institutions could select the best treatment for the patient.

The final scenario showed a substantial correlation between these five factors and patient treatment (*p* < 0.05). In the last situation, the majority (85.2%) of individuals who reported personal use of PPIs at least once a week were uninformed of the proper patient treatment. In the final scenario, almost half of the participants (49.1%) who spent less than 25% of their time on patient care could effectively select the patient’s treatment (Table 3).

**TABLE 3:** Management of the patient scenarios and perceived effectiveness of PPIs.

Variable	Categories	Scenario 1			Scenario 2			Scenario 3			Scenario 4		
		False response (n = 76)	Right response (n = 397)	*p*-value	False response (n = 96)	Right response (n = 377)	*p*-value	False response (n = 402)	Right response (n = 71)	*p*-value	False response (n = 361)	Right response (n = 112)	*p*-value
		n (%)	n (%)		n (%)	n (%)		n (%)	n (%)		n (%)	n (%)	
Age	25–29	57 (14.7)	330 (85.3)	0.133	76 (19.6)	311 (80.4)	0.538	333 (86)	54 (14)	0.204	296 (76.5)	91 (23.5)	0.960
	30–39	17 (27)	46 (73)		17 (27)	46 (73)		49 (77.8)	14 (22.2)		45 (71.4)	18 (28.6)	
	40–49	1 (5.6)	17 (94.4)		3 (16.7)	15 (83.3)		16 (88.9)	2 (11.1)		16 (88.9)	2 (11.1)	
	50–60	1 (20)	4 (80)		0 (0)	5 (100)		4 (80)	1 (20)		4 (80)	1 (20)	
Gender	Male	44 (17.8)	203 (82.2)	0.280	52 (21.1)	195 (78.9)	0.669	209 (84.6)	38 (15.4)	0.812	187 (75.7)	60 (24.3)	0.743
	Female	32 (14.2)	194 (85.8)		44 (19.5)	182 (80.5)		193 (85.4)	33 (14.6)		174 (77)	52 (23)	
Training level	Residency	52 (13.2)	342 (86.8)	<0.001*	73 (18.5)	321 (81.5)	0.033*	344 (87.3)	50 (12.7)	0.001*	304 (77.2)	90 (22.8)	0.321
	Non-trainee physician	6 (28.6)	15 (71.4)		6 (28.66)	15 (71.4)		16 (76.2)	5 (23.8)		16 (76.2)	5 (23.8)	
	Fellowship	18 (31)	40 (69)		17 (29.3)	41 (70.7)		42 (72.4)	16 (27.6)		41 (70.7)	17 (29.3)	
See outpatients in clinic	No	17 (23.3)	56 (76.7)	0.068	31 (42.5)	42 (57.5)	<0.001*	51 (69.9)	22 (30.1)	<0.001*	37 (50.7)	36 (49.3)	<0.001*
	Yes	59 (14.8)	341 (85.3)		65 (16.3)	335 (83.8)		351 (87.8)	49 (12.3)		324 (81)	76 (19)	
Patients seen per week	None	10 (22.7)	34 (77.3)	0.581	21 (47.7)	23 (52.3)	<0.001*	34 (77.3)	10 (22.7)	0.121	22 (50)	22 (50)	0.018*
	1–25	19 (17.3)	91 (82.7)		25 (22.7)	85 (77.3)		88 (80)	22 (20)		81 (73.6)	29 (26.4)	
	26–50	11 (10.6)	93 (89.4)		19 (18.3)	85 (81.7)		96 (92.3)	8 (7.7)		90 (86.5)	14 (13.5)	
	51–75	10 (14.7)	58 (85.3)		10 (14.7)	58 (85.3)		58 (85.3)	10 (14.7)		58 (85.3)	10 (14.7)	
	76–100	22 (21.6)	80 (78.4)		17 (16.7)	85 (83.3)		85 (83.3)	17 (16.7)		71 (69.6)	31 (30.4)	
	>100	4 (8.9)	41 (91.1)		4 (8.9)	41 (91.1)		41 (91.1)	4 (8.9)		39 (86.7)	6 (13.3)	
Patients seen per week on PPI	None	8 (18.6)	35 (81.4)	0.338	19 (44.2)	24 (55.8)	<0.001*	35 (81.4)	8 (18.6)	0.051	25 (58.1)	18 (41.9)	0.071
	1–25	52 (16.8)	258 (83.2)		63 (20.3)	247 (79.7)		258 (83.2)	52 (16.8)		242 (78.1)	68 (21.9)	
	26–50	12 (13.5)	77 (86.5)		11 (12.4)	78 (58.6)		81 (91)	8 (9)		69 (77.5)	20 (22.5)	
	51–75	2 (7.4)	25 (92.6)		1 (3.7)	26 (96.3)		26 (96.3)	1 (3.7)		23 (85.2)	4 (14.8)	
	76–100	1 (33.3)	2 (66.7)		1 (33.3)	2 (66.7)		2 (66.7)	1 (33.3)		2 (66.7)	1 (33.3)	
	>100	1 (100)	0 (0)		1 (100)	0 (0)		0 (0)	1 (100)		0 (0)	1 (100)	
Time spent for patient care	<25%	16 (28.1)	41 (71.9)	0.004*	25 (43.9)	32,956.1)	<0.001*	39 (68.4)	18 (31.6)	<0.001*	29 (50.9)	28 (49.1)	0.008*
	25%–49%	23 (18.1)	104 (81.9)		32 (25.2)	95 (74.8)		105 (82.7)	22 (17.3)		97 (76.4)	30 (23.6)	
	50%–74%	30 (14)	185 (86)		32 (14.9)	183 (85.1)		190 (88.4)	25 (11.6)		183 (85.1)	32 (14.9)	
	75%–100%	7 (9.5)	67 (90.5)		7 (9.5)	67 (90.5)		68 (91.9)	6 (8.1)		52 (70.3)	22 (29.7)	
Practice type	Military or other government-employed	0 (0)	5 (100)	0.151	0 (0)	5 (100)	0.001*	5 (100)	0 (0)	0.011*	5 (100)	0 (0)	0.497
	Group practice	1 (5.3)	18 (94.7)		3 (15.8)	16 (84.2)		16 (84.2)	3 (15.8)		13 (68.4)	6 (31.6)	
	Independent Practice	8 (24.2	25 (75.8)		8 (24.2)	25 (75.8)		27 (81.8)	6 (18.2)		22 (66.7)	11 (33.3)	
	Hospital employed—Government health system	28 (12.5)	196 (87.5)		29 (12.9)	195 (87.1)		204 (91.1)	20 (8.9)		181 (80.8)	43 (19.2)	
	Hospital employed—Private health system	8 (38.1)	13 (61.9)		8 (38.1)	13 (61.9)		14 (66.7)	7 (33.3)		14 (66.7)	7 (33.3)	
	Academic practice	31 (18.1)	140 (81.9)		48 (28.1)	123 (71.9)		136 (79.5)	35 (20.5)		126 (73.7)	45 (26.3)	
Familiar with guidelines on the appropriate use of PPIs to prevent upper GI bleeding	No	33 (19.4)	137 (80.6)	0.138	38 (22.4)	132 (77.6)	0.405	140 (82.4)	30 (17.6)	0.230	129 (75.9)	41 (24.1)	0.867
	Yes	43 (14.2)	260 (85.8)		58 (19.1)	245 (80.9)		262 (86.5)	41 (13.5)		232 (76.6)	71 (23.4)	
Decision support is available to help with the appropriate continuation or discontinuation of PPIs	No	50 (19.4)	208 (80.6)	0.032*	60 (23.3)	198 (76.7)	0.080	208 (80.6)	50 (19.4)	0.004*	181 (70.2)	77 (29.8)	0.001*
	Yes	26 (12.1)	189 (87.9)		36 (16.7)	179 (83.3)		194 (90.2)	21 (9.8)		180 (83.7)	35 (16.3)	
Do you personally take a PPI at least once a week?	No	58 (17.5)	273 (82.5)	0.189	77 (23.3)	254 (76.7)	0.014*	273 (82.5)	58 (17.5)	0.020*	240 (72.5)	91 (27.5)	0.003*

### 3.5 Predictors of appropriate management in the GERD scenario

Patient management in the GERD scenario was significantly related to the degree of training (*p*-value <0.05). In the first scenario, non-trained physicians had a lower chance of successfully managing the patient than did resident doctors. (*p*-value = 0.001; OR = 0.015).

Participants who reported spending 75%–100% of their time caring for patients were 5.33 times more likely to manage the patient effectively in the GERD scenario than those who reported spending less than 25% of their time caring for patients (*p*-value = 0.020) (Table 4).

**TABLE 4:** Predictors of appropriate management in the GERD scenario.

Variable	Categories	Scenario 1		
		AOR	95%CI for B	*p*-value
Age	25–29	Ref		
	30–39	1.381	0.455/4.189	0.569
	40–49	–		
	50–60			
Gender	Male	Ref		
	Female	1.388	0.783/2.461	0.261
Training level	Residency	Ref		
	Non-trainee physician	0.015	0.001/0.187	0.001
	Fellowship	0.261	0.079/0.866	0.028
See outpatients in clinic	No	Ref		
	Yes	1.772	0.669/4.694	0.249
Patients seen per week	None	Ref		
	1–25	1.285	0.238/6.937	0.771
	26–50	1.798	0.303/10.675	0.519
	51–75	1.440	0.212/9.790	0.710
	76–100	0.797	0.114/5.599	0.820
	>100	0.920	0.097/8.723	0.942
Patients seen per week on PPI	None	Ref		
	1–25	0.382	0.081/1.805	0.225
	26–50	0.600	0.106/3.399	0.564
	51–75	1.055	0.110/10.087	0.963
	76–100	0.281	0.012/6.836	0.436
	>100	–		
Time spent on patient care	<25%	Ref		
	25%–49%	1.797	0.704/4.586	0.220
	50%–74%	2.719	0.998/7.410	0.051
	75%–100%	5.336	1.303/21.856	0.020
Practice type	Military or other government employed	Ref		
	Group practice	–		
	Independent practice			
	Hospital employed—Government health system			
	Hospital employed—Private health system			
	Academic practice			
Familiar with guidelines on appropriate use of PPIs to prevent upper GI bleeding	No	Ref		
	Yes	1.306	0.696/2.449	0.406
Decision support available to help with appropriate continuation or discontinuation of PPIs	No	Ref		
	Yes	1.613	0.824/3.159	0.163
Do you personally take a PPI at least once a week?	No	Ref		
	Yes	1.214	0.609/2.422	0.582

### 3.6 Strategies to reduce the adverse effects of PPIs

Participants who reported frequently\sometimes reducing their daily PPI dosage from a normal dose to half a standard dose to decrease side effects were 55.6%. Half of the participants (52.4%) said they never relied on reducing the daily dosage of PPIs to prevent adverse effects. One-third of participants (31.5%) said that they frequently\sometimes stop daily PPIs and prescribed an H2-blocker for the first few weeks to minimize rebound symptoms. Only (36.6%) said they frequently\sometimes quit using PPIs to avoid side effects (Table 5).

**TABLE 5:** Strategies to reduce the adverse effects of PPIs.

Variable	Categories	N (%)
Reduce daily PPI dose from a standard dose to half of a standard dose (e.g., omeprazole 10 mg daily)	Frequently	77 (16.3)
	Sometimes	186 (39.3)
	Occasionally	152 (32.1)
	Never	58 (12.3)
Substitute daily PPI with a daily H2-blocker	Frequently	69 (14.6)
	Sometimes	191 (40.4)
	Occasionally	169 (35.7)
	Never	44 (9.3)
Slowly taper a daily PPI.	Frequently	12 (2.5)
	Sometimes	61 (12.9)
	Occasionally	152 (32.1)
	Never	248 (52.4)
Stop daily PPI and prescribe an H2-blocker for the first few weeks after discontinuation to prevent rebound symptoms	Frequently	16 (3.4)
	Sometimes	133 (28.1)
	Occasionally	241 (51)
	Never	83 (17.5)
Simply stop the PPI.	Frequently	31 (6.6)
	Sometimes	142 (30)
	Occasionally	213 (45)
	Never	87 (18.4)

### 3.7 The mean score ±SD of consideration of bone fracture risk and GERD symptoms recurrences in the first scenario

In the first scenario, eight of 11 factors (*p*-value <0.05) were substantially related to GERD symptom recurrence. Participants who were familiar with the guidelines on properly using PPIs to avoid upper GI bleeding performed better when evaluating GERD symptom recurrence (5.01 ± 1.052 versus 4.59 ± 1.412). The mean score for evaluating GERD symptoms among those caring for outpatients was more significant (4.96 ± 1.152) than among those who did not (4.32 ± 1.373).

In the first scenario, eight factors were significantly associated with bone fracture risk with respect to patient treatment (*p* < 0.05). Respondents aged 25–29 years scored higher than other age groups when assessing the risk of bone fractures (5.02 ± 1.215). Participants who did not personally use PPI at least once a week had a higher mean score when considering bone fractures (5.04 ± 1.277). Participants who did not have access to decision assistance to assist with appropriate PPI continuation or termination had a higher score for bone fracture risk (5.05 ± 1.317) than those who did (4.90 ± 1.230). Compared to other training groups, resident physicians scored better when evaluating bone fracture risk (5.07 ± 1.189) (Table 6).

**TABLE 6:** The mean score ±SD of consideration bone fracture risk and GERD symptoms recurrences in the first scenario and upper GI bleeding risk and bone fracture risk in the fourth scenario.

Variable	Categories	Scenario 1		Scenario 4	
		Recurrence of her GERD symptoms	Consideration of bone fracture risk	Consideration of upper GI bleeding risk	Consideration of bone fracture risk
		(Mean ± SD)	(Mean ± SD)	(Mean ± SD)	(Mean ± SD)
Age	25–29	4.77 ± 1.195*	5.02 ± 1.215*	5.45 ± 0.936	4.08 ± 1.006*
	30–39	5.21 ± 1.297	4.78 ± 1.570	5.40 ± 1.056	3.81 ± 1.229
	40–49	5.50 ± 0.857	4.89 ± 1.568	5.44 ± 0.705	3.83 ± 1.150
	50–60	5.00 ± 1.000	5.00 ± 1.00	5.80 ± 0.447	4.00 ± 1.225
Gender	Male	4.92 ± 1.190	4.89 ± 1.382	5.45 ± 0.895*	4.01 ± 1.048
	Female	4.79 ± 1.229	5.08 ± 1.150	5.45 ± 0.989	4.06 ± 1.048
Training level	Residency	4.76 ± 1.191*	5.07 ± 1.189*	546 ± 0.933	4.11 ± 1.003*
	Non-trainee physician	5.14 ± 1.352	4.52 ± 1.662	5.52 ± 0.680	3.62 ± 1.117
	Fellowship	5.40 ± 1.138	4.52 ± 1.570	5.33 ± 1.066	3.69 ± 1.217
See outpatients in clinic	No	4.32 ± 1.373*	4.68 ± 1.480	5.12 ± 1.322*	3.68 ± 1.257*
	Yes	4.96 ± 1.152	5.03 ± 1.233	5.51 ± 0.841	4.10 ± 0.993
Patients seen per week	None	4.27 ± 1.404*	4.52 ± 1.649*	4.98 ± 1.486*	3.48 ± 1.338*
	1–25	4.61 ± 1.142	4.89 ± 1.207	5.32 ± 1.108	3.95 ± 0.999
	26–50	4.84 ± 1.080	4.93 ± 1.082	5.54 ± 0.762	3.95 ± 0.969
	51–75	4.99 ± 1.321	5.21 ± 1.241	5.53 ± 0.762	4.32 ± 0.984
	76–100	5.25 ± 1.059	4.93 ± 1.388	5.54 ± 0.699	4.10 ± 1.000
	>100	4.98 ± 1.288	5.51 ± 1.079	5.71 ± 0.727	4.38 ± 0.984
Patients seen per week on PPI	None	4.44 ± 1.385	4.67 ± 1.584	5.19 ± 1.367	3.72 ± 1.297*
	1–25	4.92 ± 1.131	4.95 ± 1.210	5.45 ± 0.894	4.01 ± 0.995
	26–50	4.85 ± 1.293	5.12 ± 1.269	5.53 ± 0.813	4.18 ± 1.051
	51–75	4.85 ± 1.262	5.56 ± 0.974	5.74 ± 0.526	4.48 ± 0.802
	76–100	5.67 ± 0.577	4.00 ± 2.646	5.67 ± 0.577	4.00 ± 1.732
	>100	1.00 ± 0	1.00 ± 0	1.00 ± 0	1.00 ± 0
Time spent on patient care	<25%	4.35 ± 1.408*	4.61 ± 1.656*	5.05 ± 1.493*	3.49 ± 1.241*
	25%–49%	4.50 ± 1.201	4.76 ± 1.277	5.38 ± 0.959	3.87 ± 1.042
	50%–74%	5.18 ± 1.062	5.04 ± 1.205	5.53 ± 0.716	4.16 ± 0.978
	75%–100%	4.92 ± 1.191	5.46 ± 0.982	5.66 ± 0.848	4.38 ± 0.887
Practice type	Military or other government-employed	5.20 ± 1.304*	4.40 ± 1.673*	520 ± 0.837	3.80 ± 0.837
	Group practice	4.37 ± 1.165	5.21 ± 0.918	4.89 ± 1.560	4.21 ± 1.182
	Independent practice	5.12 ± 0.229	5.06 ± 1.580	5.79 ± 0.893	3.97 ± 1.262
	Hospital employed—Government health system	4.53 ± 1.220	5.03 ± 1.183	5.42 ± 0.900	4.03 ± 0.951
	Hospital employed—Private health system	4.53 ± 1.220	4.19 ± 1.601	5.38 ± 1.244	3.33 ± 1.278
	Academic practice	5.26 ± 1.027	4.99 ± 1.297	5.50 ± 0.850	4.13 ± 1.060
Familiar with guidelines on the appropriate use of PPIs to prevent upper GI bleeding	No	4.59 ± 1.412*	5.06 ± 1.400*	5.39 ± 1.027	4.13 ± 1.134*
	Yes	5.01 ± 1.052	4.93 ± 1.205	5.49 ± 0.887	3.98 ± 0.993
Decision support is available to help with the appropriate continuation or discontinuation of PPIs	No	4.74 ± 1.325*	5.05 ± 1.317*	5.48 ± 0.971	4.08 ± 1.079*
	Yes	4.99 ± 1.041	4.90 ± 1.230	5.42 ± 0.903	3.98 ± 1.007
Do you personally take a PPI at least once a week?	No	4.79 ± 1.275	5.04 ± 1.277*	5.41 ± 0.982	4.08 ± 1.049*
	Yes	5.01 ± 1.028	4.84 ± 1.275	5.54 ± 0.831	3.92 ± 1.039

*p-value < 0.05.

### 3.8 The mean score ±SD of consideration of upper GI bleeding risk and bone fracture risk in the fourth scenario

In the end scenario, four out of 11 factors (*p*-value <0.05) were associated with the risk of upper GI bleeding during patient treatment. Participants who cared for more than 100 patients per week had a higher score in considering GI bleeding risk than those who cared for fewer patients (5.71 ± 0.727), and those who cared for outpatients had a higher score in evaluating upper GI bleeding risk than those who did not (5.51 ± 0.841 versus 5.12 ± 1.322).

The last scenario showed a significant association between the nine factors and bone fracture risk (*p*-value <0.05). Respondents familiar with guidelines on the appropriate use of PPIs to prevent upper GI bleeding scored lower when considering bone fracture risk than those unfamiliar (3.98 ± 0.993 versus 4.13 ± 1.134). Participants who took a PPI at least once a week had a lower score when considering bone fracture risk (3.92 ± 1.039) than those who did not (4.08 ± 1.049). When comparing bone fracture risk to other age groups, the age group 25-29 years had the highest score (4.08 ± 1.006). The mean score for considering bone fracture risk among outpatients was greater than that among those who did not (4.10 ± 0.993 versus 3.68 ± 1.257, respectively) (Table 6).

## 4 Discussion

Proton pump inhibitors (PPIs) are a class of medications used to treat acid-related disorders, such as gastroesophageal reflux disease, peptic ulcers, and other diseases, and they must be used following the most recent guidelines because unnecessary long-term use of these medications has been linked to the occurrence of adverse effects (Fossmark et al., 2019). The appropriate use of these drugs is based on the correct indication from the clinician, who should have proper updated knowledge about the indications and side effects associated with these drugs, which complies with the aim of this study to determine the physicians’ perceptions about the adverse effects of PPIs with prescribing and deprescribing attitudes (Scarpignato et al., 2016).

In this study of Syrian physicians, 65.5% of the respondents followed up with one to 25 patients taking PPIs per week, and 64.1% were familiar with the PPI guidelines, indicating that a large percentage were involved in the care of these patients according to the updated guidelines. However, there is a gap in the percentage of those who are aware of the published data on the adverse effects associated with PPI use. Based on the detrimental effects, 33.2% of respondents had very much modified their behaviors in prescribing PPIs, indicating a possible practice gap that might result in an incorrect judgment of de-prescribing PPIs. A small percentage of respondents (11.8%) confirmed that they frequently discuss the potential adverse effects of PPIs with patients, which is a deficient percentage that may lead to a false perception among patients that PPIs are safe drugs that can be used on a long term basis without any concerns, and this is considered a risky habit, especially for the elderly people who have multiple comorbidities and are vulnerable to drug-drug interactions (Schonheit et al., 2016).

There was a significant correlation between the appropriate management of the GERD patient and the training level and time spent caring for the patients. There was a difference in the physicians’ attitude regarding the PPI prescription to the risk of PPI adverse effects, with 39.3% sometimes intending to decrease the daily dose of the PPIs, 40.4% planning to sometimes substitute the PPIs with H2 blockers, and 52.4% stating that they never intend to tap the daily dose of PPIs. A study that evaluated the intention of clinicians in four medical and surgical specialties to prescribe PPIs according to the guidelines for the prevention of upper gastrointestinal bleeding using a semi-structured questionnaire found that each faced distinct barriers to PPI gastroprotection use related to knowledge, decision processes, and professional roles (Kurlander et al., 2022).

A study conducted in Japan to identify the prescription pattern of PPIs among hemodialysis patients found that 60% of physicians intend to continue prescribing PPIs in hemodialysis patients. In contrast, others may be depressed due to the risk of adverse effects associated with using PPIs. This is consistent with our findings that physicians are aware of the guidelines regarding PPIs, and that upper gastrointestinal bleeding is fearful of deviating from them (Kawarazaki et al., 2020). Our results showed that 48.9% of the respondents were concerned about the PPI-associated adverse effects when prescribing them; however, a study conducted in the United States to examine the concern and awareness of patients receiving PPIs regarding the adverse effects associated with them reported that 32% of the patients were somewhat concerned about the adverse effects (Kurlander et al., 2019).

Another study conducted in a medical center in the USA involved a structured survey of clinicians from different specialties to assess their intention to prescribe PPIs in the context of their associated risk factors. It was reported that 80% of the gastrointestinal clinicians discuss the benefit-risk ratio of the PPIs with the patients, 21% of them intend to decrease the dose of PPI, and 13% plan to substitute the PPIs with the H2 Blockers, which is consistent with the results of our study that 14.6% intend to frequently replace the PPIs with the H2 blockers (Al-Qaisi et al., 2018).

In another prospective study (Calvo et al., 2021), which involved gastroenterology patients on chronic use of PPIs, 86 patients out of a total of 285 patients were on unnecessary PPI therapy, and 75 of them accepted the deprescribing algorithm that was applied to them. The patients were followed up for 4 weeks, 12, and 24 weeks to determine if they returned to using the drugs, and it was observed that the number of patients decreased with each follow-up period, which indicates that there is a high percentage of patients who are on chronic PPI use are using them inappropriately.

In a study that examined the inappropriate use of PPIs in an outpatient clinic found that 35.9% of the total sample were using the PPIs without a correct indication for that, which was more common among geriatric patients over the age of 60 years. The most common reason was that they continued to use medication due to the lack of follow up, which is considered high risk because the absence of a discussion between the patient and the clinician may show a false impression about the drugs, as shown in the results of this study (Çelik et al., 2021).

This study indicates that there is a gap between clinicians and patients that needs to be filled for better health outcomes by following the proper discussion between the two parties to find the most suitable drugs according to the patient’s case, and the clinician should evaluate the case precisely before prescribing PPIs for prevention.

Clinicians should follow the updated AGA guidelines regarding the prescription and de-prescription of proton pump inhibitors (PPIs), which state that the PPI prescription should be based on proper indication for the PPIs in the appropriate patient by evaluating the case with reasonable tests to identify any risk factors or complications that are present, such as erosive and eosinophilic esophagitis. As the patient will not be a candidate for deprescribing in these circumstances, the patient should undergo a reasonable assessment to determine whether the rationale is still present, as this should be the foundation for deprescribing PPIs (Targownik et al., 2022). Moreover, future research should include a large sample size of physicians from different Arab and non-Arab countries to assess any gaps and take proper action towards lessen any gaps found.

### 4.1 Limitations and strengths

Among the strengths of the study is that a reasonable sample of clinicians responded to the survey, which is well-structured and developed, as demonstrated in the Methods section. In addition, insufficient data are available to determine Syrian clinicians’ perspectives on PPI AEs. This is the first study to assess Syrian clinicians’ perspectives on PPI AEs, the benefits of lowering UGIB, and how these perceptions are related to PPI prescription practices. The large sample size in this study augments the statistical power and confirms the generalizability of the results. However, the limitations include that the responses in the clinical scenario may be influenced by previous questions about the adverse effects of PPIs, which may differ from clinical practice when dealing with the same scenarios. Moreover, regions with inadequate access to electricity or Internet connectivity were deprived of this questionnaire administration. In addition, the number of physicians’ years of experience was not measured, which may have affected their knowledge and judgments.

## 5 Conclusion

This study revealed a gap in Syrian physicians’ knowledge and practice of PPI use. Even Although 64.1% of the participants were familiar with the PPI guidelines, only a small percentage were aware of the published data on the adverse effects associated with PPI use of PPIs. Only a percentage of 11.8% reported that they frequently discussed the potential PPIs AEs with the patients. Meanwhile, there was a significant correlation between the appropriate management of the GERD patient and the training level and time spent caring for the patients. These results highlight the importance of equipping Syrian physicians with updated knowledge and skills regarding PPI usage and encouraging doctor-patient discussions to ensure proper PPI usage with appropriate duration and minimize AEs.

## Data Availability

The raw data supporting the conclusion of this article will be made available by the authors, without undue reservation.
