# Supplementary material for: Evaluating physicians’ awareness and prescribing trends regarding proton pump inhibitors: a cross-sectional study
Source: Front Pharmacol. 2023 Nov 9;14:1241766. doi: 10.3389/fphar.2023.1241766 (PMC10665834; doi:10.3389/fphar.2023.1241766)
Supplement: Supplementary file 1 [file DataSheet1.PDF]

Thank you for your willingness to participate in this survey. We are interested in learning more about how you use proton pump inhibitors (PPIs) in your clinical practice. PPIs include medications such as omeprazole (Prilosec), esomeprazole (Nexium), pantoprazole (Protonix), and lansoprazole (Prevacid).

1. **How familiar, if at all, are you with the published scientific data on possible adverse effects from PPI use?**

   Very familiar
   Somewhat familiar
   Slightly familiar
   Not at all familiar Skip to Q. 3

2. **How much, if at all, have recent studies about adverse effects of proton pump inhibitors (PPIs) changed your PPI prescribing habits?**

   Very much
   Somewhat
   Slightly
   Not at all

3. **In general, how concerned are you about adverse effects when prescribing PPIs to your patients?**

   Very much
   Somewhat
   Slightly
   Not at all

4. **How frequently, if ever, do you discuss the risks of adverse effects with patients before starting a PPI?**

   Often
   Sometimes
   Rarely
   Never

5. **How frequently, if ever, do patients on PPI therapy bring up concerns about the risk of adverse effects from PPIs?**

   Often

Sometimes
Rarely
Never

6. **Do you believe PPIs <u>increase the risk</u> of any of the following conditions?**

	**Yes**	**No**	**Unaware of association with PPI use**
Acute Interstitial Nephritis			
Chronic Kidney Disease			
Clostridium Difficile infection			
Death			
Dementia			
Fracture of a bone			
Gastric cancer			
Heart Attack			
Osteoporosis or Osteopenia (weakening of the bones)			
Pneumonia			
Stroke			
Vitamin B12 deficiency			
Vitamin D deficiency			

[PAGE BREAK. Do not allow participants to go back to an earlier page]

7. **(Only show the conditions from Q.6 to which Respondent said "yes". If "None", Skip to first scenario) When you prescribe PPIs, which one of these possible adverse effects do you worry <u>most</u> about clinically? (select one)**

Acute Interstitial Nephritis
Chronic Kidney Disease
Clostridium Difficile infection
Dementia
Fracture of a Bone
Gastric Cancer
Heart Attack
Osteoporosis or Osteopenia (Weakening of the Bones)
Pneumonia
Stroke
Vitamin B12 Deficiency
Vitamin D Deficiency
None of these (Exclusive response)

[PAGE BREAK. Do not allow participants to go back to an earlier page]

We will now present four different scenarios. In each one, the patient defers to you for management of her medications.

(Randomize the first three scenarios. The 4th one will always be last)

Scenario 1 Minimal GI bleed risk

A 70 year-old woman presents to your clinic for routine follow-up. She has a prior history of gastroesophageal reflux disease (GERD) (heartburn several times a week), now with no reflux symptoms on omeprazole 20 mg daily for many years. Her medical history is otherwise unremarkable. She takes no other medications. Recent laboratory studies and an upper endoscopy are unremarkable. She recently underwent a Dual Energy X-ray Absorptiometry (DEXA) scan, which revealed osteopenia.

8. How would you manage the patient's omeprazole?

Continue the omeprazole
Stop the omeprazole
Stop the omeprazole and also start an H2-blocker

9. How important, if at all, would preventing recurrence of her GERD symptoms be to your decision?

Not at all important 0	1	2	3	4	5	Extremely important 6
○	○	○	○	○	○	○

10. How important, if at all, would consideration of bone fracture risk be in your decision?

Not at all important 0	1	2	3	4	5	Extremely important 6
○	○	○	○	○	○	○

Scenario 2 **(New Page) Low GI bleed risk**

A 70 year-old woman presents to your clinic for routine follow up. She has a history of coronary artery disease and atrial fibrillation for which she takes aspirin 81 mg daily and warfarin. She also takes omeprazole 20 mg daily to "protect her stomach." She has no history of gastrointestinal problems. She takes no other medications and has no symptoms. She recently underwent a Dual Energy X-ray Absorptiometry (DEXA) scan, which revealed osteopenia.

11. **How would you manage the patient's omeprazole?**

Continue the omeprazole
Stop the omeprazole
Stop the omeprazole and also start an H2-blocker

Scenario 3 **(New Page) Moderate GI bleed risk**

A 70 year-old woman presents to your clinic for routine follow up. She takes aspirin 81 mg daily for a history of coronary artery disease. She also takes omeprazole 20 mg daily to "protect her stomach." She has no history of gastrointestinal problems. She takes no other medications and has no symptoms. She recently underwent a Dual Energy X-ray Absorptiometry (DEXA) scan, which revealed osteopenia.

12. **How would you manage the patient's omeprazole?**

Continue the omeprazole
Stop the omeprazole
Stop the omeprazole and also start an H2-blocker

Scenario 4 **(High GI bleed risk)(logistic regression)**

A 70 year-old woman presents to your clinic for routine follow-up. She has a history of coronary artery disease for which she takes aspirin 81 mg daily. About 10 years ago, she underwent an endoscopy due to dyspepsia and was diagnosed with peptic ulcer disease caused by use of ibuprofen, which she no longer uses. She takes omeprazole 20 mg daily

to "protect her stomach." She currently has no symptoms. She recently underwent a Dual Energy X-ray Absorptiometry (DEXA) scan, which revealed osteopenia.

13. **How would you manage the patient's omeprazole?**

    Continue the omeprazole
    Stop the omeprazole
    Stop the omeprazole and also start an H2-blocker

14. **How important, if at all, would consideration of upper GI bleeding risk be in your decision?**

Not at all important 0	1	2	3	4	5	Extremely important 6
○	○	○	○	○	○	○

15. **How important, if at all, would consideration of bone fracture risk be in your decision?**

Not at all important 0	1	2	3	4	5	Extremely important 6
○	○	○	○	○	○	○

16. **How effective, if at all, do you believe omeprazole is at reducing the risk of upper GI bleeding in the patient in this last scenario?**

    Very effective
    Moderately effective
    Slightly effective
    Not at all effective

17. **Would your recommendation regarding omeprazole change if you knew the following information with certainty: The patient's risk of upper GI bleeding is 2.7% per year, and use of a PPI can reduce this risk to 1.1% per year. In addition, her risk of hip fracture is 1.0% per year, and omeprazole increases the risk to 1.3% per year.**

Yes
No Skip to Q. 19

18. What would your new recommendation be?

Continue the omeprazole
Stop the omeprazole
Stop the omeprazole and also start an H2-blocker

New Page

19. How often, if ever, have you used each of the following strategies with your patients because you were concerned about long term PPI harms?

	**Never**	**Occasionally**	**Sometimes**	**Frequently**
Recommend using PPI only on-demand/as needed instead of daily				
Reduce daily PPI dose from a standard dose to half of a standard dose (e.g., omeprazole 10 mg daily)				
Substitute daily PPI with a daily H2-blocker (e.g., Zantac)				
Slowly taper a daily PPI				
Stop daily PPI, and prescribe an H2-blocker (e.g., Zantac) for the first few weeks after discontinuation to prevent rebound symptoms				
Simply stop the PPI				

Page Break

The following questions will tell us a little about you and your current practice environment.

20. What is your age?

Drop down box

21. What is your gender?

Male
Female
Other (Specify: ____)
Prefer not to answer

22. Please indicate your current position (Select one)

In a residency training program	Skip to Q. 27
In a fellowship training program	Skip to Q. 27
Attending physician	

23. Which of the following best describes you?

Internal Medicine specialist (i.e., specialize in general internal medicine or hospital medicine)
Subspecialist in gastroenterology
Subspecialist noninvasive cardiology
Subspecialist invasive cardiology
Other medical subspecialist

24. In what year did you finish your residency in internal medicine?

----

25. (If Subspecialist) In what year did you finish fellowship?

____

26. Are you currently board certified in your specialty?

Yes
No

27. Do you currently see patients in an outpatient clinic?

Yes
No

28. In a typical week, how many patients do you see in your practice?

None
1 - 25
26 – 50
51 – 75
76 – 100
.> 100

29. In a typical week, how many patients <u>who take a PPI</u> do you see in your practice?

None
1 - 25
26 – 50
51 – 75
76 – 100
.> 100

30. Roughly what percentage of your workweek do you spend on direct patient care?

< 25%
25% - 49%|
50% - 74%
75% - 100%

31. How would you best describe your practice setting?

Solo practice
Group practice
Academic practice (affiliated with a medical school)

Veterans Affairs health system
Military or other government employed
Hospital employed – Integrated health system (e.g., Kaiser Permanente)
Hospital employed – Private health system

32. Are you familiar with any guidelines or professional recommendations on when it is appropriate to use PPIs for the prevention of upper GI bleeding?

Yes
No

33. Does your practice have any decision support systems in place to help you evaluate when it is appropriate to continue or discontinue a patient's PPI?

Yes
No

34. Do you personally take a PPI at least once a week?

Yes
No
Prefer not to answer

Thank you for taking the time to complete this survey.
